# A TIR Domain Protein from *E. faecalis* Attenuates MyD88-Mediated Signaling and NF-κB Activation

**DOI:** 10.1371/journal.pone.0112010

**Published:** 2014-11-04

**Authors:** Jun Zou, Arto S. Baghdayan, Sarah J. Payne, Nathan Shankar

**Affiliations:** Department of Pharmaceutical Sciences, University of Oklahoma Health Sciences Center, Oklahoma City, Oklahoma, United States of America; University of Kansas, United States of America

## Abstract

Toll-like receptor signaling, mediated by functional Toll/interleukin-1 receptor (TIR) domains, plays a critical role in activating the innate immune response responsible for controlling and clearing infection. Bacterial protein mimics of components of this signaling pathway have been identified and function through inhibition of interactions between Toll-like receptors (TLRs) and their adaptor proteins, mediated by TIR domains. A previously uncharacterized gene, which we have named *tcpF* (for TIR domain-containing protein in *E. faecalis*) was identified in the genome of *Enterococcus faecalis V583*, and predicted to encode a protein resembling mammalian and bacterial TIR proteins. We overexpressed and purified TcpF from *E. coli* and found that the recombinant protein could bind to phosphatidylinositol phosphates *in vitro*, suggesting a mechanism by which TcpF may be anchored to the plasma membrane in close proximity to TIR domains of TLRs and adaptor proteins. Purified TcpF was also found to interact specifically with the TIR adaptor protein MyD88, and this interaction was dependent on the BB loop domain in the Box 2 region of TcpF. Despite no evidence of TcpF being a secreted protein, recombinant TcpF was effectively able to enter RAW264.7 cells *in vitro* although the mechanism by which this occurs remains to be determined. Overexpression of TcpF in mammalian cells suppressed the NF-κB activation induced by bacterial lipoteichoic acid. A mutant lacking the *tcpF* gene was attenuated for survival in macrophages, with increased ability to activate NF-κB compared to the wild type strain. Complementation in *trans* restored growth, and inhibition of NF-κB, to that of wild type levels. No appreciable difference in bacterial persistence, dissemination or pathogenesis was observed between the wild type and mutant in a mouse peritonitis model however, which suggested either a subtle role for TcpF or functional overlap with other redundant factor(s) in this virulence model.

## Introduction

*Enterococcus faecalis* is a Gram-positive, catalase-negative, non-spore forming, facultative anaerobe that can occur as single cocci or in chains and normally resides as a part of the commensal flora in the gastrointestinal tract of mammals [1]. Although enterococci rarely cause infections in the healthy host; in immunocompromised patients or individuals on antibiotic therapy, these organisms can cause serious disease including urinary tract infections, hepatobiliary sepsis, endocarditis, surgical wound infections, bacteremia, and neonatal sepsis [2]. For enterococci to cause infections it must be able to survive the host defense mechanisms, such as those encountered during translocation from the gastrointestinal tract into the peritoneal cavity. While significant strides have been made in recent years regarding enterococcal virulence factors, much remains to be learned about the interactions of this opportunistic pathogen with the host.

The first line of defense against bacterial infections is provided by the innate immune system consisting of macrophages, neutrophils, and dendritic cells [3]. These cells directly kill the microorganisms through phagocytosis or induce production of inflammatory cytokines. The Toll-like receptors (TLRs) have a central role in pathogen detection and initiate quick innate immune response to control many microorganisms during infection [4]. A central conserved feature of this TLR signaling pathway is the Toll/interleukin-1 receptor (TIR) domain which is found in both the cytoplasmic regions of TLRs and in adaptor proteins including MyD88, TIRAP, TRAM, TRIF, and SARM [5]. The recognition of cognate ligands by TLRs induces a conformational change in the cytoplasmic TIR domains and activates signaling cascades which result in the production of innate effector responses as well as the initiation of an adaptive immune response [6]. The adaptor proteins are key to this process and are recruited to the cytoplasmic domains of the TLRs via TIR-TIR interactions. The adaptors (with the exception of SARM) recruit downstream signaling molecules (IRAK, TAK and IKK) leading to the activation of NF-κB and members of the IRF family of transcription factors. This results in the production of pro-inflammatory cytokines and type-1 interferons [7], [8].

Bacterial pathogens produce a variety of virulence factors that interact with host molecules and modulate immune defense processes including phagocytosis, signal transduction, cytoskeletal rearrangements and cytokine production [9]–[13]. These factors may be anchored to the bacterial cell surface, injected into the host cell upon attachment to the host cell surface or secreted through dedicated secretion systems. Recently, a class of bacterial proteins with homology to the Toll/IL-1 receptor (TIR) domain has been identified. The first of these from *Salmonella enterica* serovar Enteritidis was named TlpA (for TIR-like protein A) [14]. Similar TIR domain-containing proteins (Tcps) have been subsequently identified and characterized, including TcpC from uropathogenic *E. coli* CFT073 [15], TcpB from *Brucella melitensis* [15], Btp1 from *Brucella abortus* [16], PdTLP, a Toll-like protein from *Paracoccus denitrificans* [17] and TirS from *Staphylococcus aureus* [18].

That bacteria could target TLR signaling to enhance virulence was demonstrated through functional characterization of TcpB and TcpC [15]. Cells infected with a TcpC-deficient mutant of CFT073 showed greater production of the proinflammatory cytokines tumor necrosis factor (TNF) and interleukin-6 (IL-6) and decreased bacterial load compared to cells infected with wild-type CFT073. Cells transfected with plasmids encoding TLR signaling components and the TcpC or TcpB proteins, along with reporter plasmids, exhibited decreased signaling through TLR4 and TLR2, coupled to the adaptor MyD88. CFT073 infection of MyD88-deficient cells did not show a decrease in TNF secretion. Thus, TcpC and TcpB appeared to selectively interfere with MyD88-dependent TLR pathways, similar to that observed for TlpA [14] and PdTLP [17]. The TIR domain of TcpC or full-length TcpB bound to MyD88 in pull-down assays. Finally, the importance of TcpC in disease was determined by infecting mice with the wild-type CFT073 or the TcpC-deficient strain, and those with the wild-type strain had more aggressive disease. Similar results were obtained in studies with TcpB from *Brucella melitensis*, found to mimic properties of the Toll-like receptor adaptor protein TIRAP [19] while Btp1 from *Brucella abortus* was found to down-modulate maturation of infected dendritic cells by interfering with the TLR2 signaling pathway [16].

In the present study, we describe the identification and characterization of an enterococcal TIR domain protein. Bioinformatic analysis of the *Enterococcus faecalis* strain V583 genome revealed a single open reading frame (EF1959) potentially encoding a 274 amino acid protein with a conserved TIR domain, of unknown function. We have now designated this protein as TcpF (for TIR-domain containing protein in *E. faecalis*). Our studies reveal that TcpF can directly interact with the adaptor protein MyD88 and can block NF-κB induction by stimuli that involve TIR domain proteins. Compared to the wild type strain, a TcpF deletion mutant showed increased ability to activate NF-κB and exhibited lower intracellular survival in infected macrophages. In addition, recombinant TcpF could be internalized by RAW264.7 cells as well as bind to a number of phosphorylated phosphoinositides (PIPs). However, no significant differences were observed between the wild type and TcpF mutant strains in systemic spreading or histopathology in a murine peritonitis infection model.

## Materials and Methods

### Bacterial strains, plasmids, and culture conditions

All strains and plasmids used in this study are listed in Table 1. *Escherichia coli* strains EC1000 and XL10-Gold were used as hosts for plasmid purification, and C41 (DE3) for protein purification. *E. faecalis* and *E. coli* strains were cultivated in brain heart infusion (BHI), Todd Hewitt broth (THB) containing 1% glucose, tryptic soy broth (TSB) contain 0.75% glucose, or Luria-Bertani (LB) broth. Antibiotics were obtained from Sigma Chemical (St. Louis, MO) and used at the following concentrations: kanamycin (25 µg/ml) for E99 and SPB03; spectinomycin (500 µg/ml) for SPB04. RAW264.7 cells and Mouse Embryonic Fibroblast (MEF) cells were cultivated initially in Dulbecco's Modified Eagle medium (DMEM) containing 4.5 g/L glucose, L-glutamine and sodium pyruvate plus 10% fetal bovine serum (FBS), to confluence in T-25 flasks. All primers used in this study are listed in Table 2.

**Table 1:** *Enterococcus faecalis* strains and plasmids used in this work.

Plasmid/Strain	Notes	Reference
pAT28	Derivative of pUC18 and pAMβ1; Spectinomycin resistance marker	[47]
pLT06	Temperature sensitive cloning vector; Derivative of pCJK47; Chloramphenicol resistance marker	[48]
pMAL-c5E	Produces maltose-binding protein fusions	[49]
pMV158GFP	Derivative of pMV158 carrying green fluorescence protein gene	[50]
pOU1811	pMAL-c5E expressing MBP-TcpF fusion	This study
pOU1812	pMAL-c5E expressing TcpF BB-loop mutant	This study
pSJP01	Construct for generating TcpF mutant	This study
V583	Clinical blood isolate; vancomycin-resistant	[30]
T1	CDC reference strain/Japan	[51]
D6	Pig isolate/Denmark	[51]
T3	Clinical urine isolate/Japan	[51]
OG1RF	Clinical oral isolate; plasmid-free	[51]
E99	Clinical blood isolate	[51]
SPB03	*tcpF*-deficient mutant of E99	This study
SPB04	*tcpF*-complement of SPB03	This study

**Table 2:** Oligonucleotide primers used in this work.

Name	Sequence 5′ to 3′
B2-F	ACGGTCGACCTGTGTTCGAAACCCAAAC
B2-R	ACGGTCGACTCCACTATTGAAACAGGTG
EF1958-F	GAACACGTTTAACACATAGCTTAGAAGTGG
EF1958-R	GAGAGAATTCGTATAGGCGATATCATCTGAAGCT
EF1958-R1	GCCTGAAAGCATTTCACTTCATCACAATCT
EF1959-F	GAGAGGATCCGCTCACTACTCAACATCCTCCAAG
EF1959-F1	GAGAGGATCCTACGTCTCTTATTTAGAGAAGGTAG
EF1959-F2	TTGAAACAGGTGTACAGCCAGGAGAATTAA
EF1959-R	GAGAGGATCCTTACTCTACCTTCTCTAAATA
EF1959-R1	GCCAATGGCTTTCATCCTCTGATAGCGTGT
EF1961-F	AAGCTGGCTACACTGCAGTTGTATCTCACC
KS05Seq-R	CCTATTATACCATATTTTGGAC
Ori-F	CAATAATCGCATCCGATTGCA

### Bioinformatics and protein structure modeling

BLAST analyses were performed using the gene and deduced protein sequences of TcpF, employing servers at the National Center for Biotechnology Information (NCBI), the Enterococcus Group Database at the Broad Institute and at the Baylor College of Medicine. Multiple protein sequence alignments were done using T:COFFEE, a tool that uses a combination of local and global alignments that leads to a significant increase in alignment accuracy and proven to be more accurate than its counterparts in a wide variety of cases [20]. Comparative structure modeling was done using 3D-JIGSAW for PDB generation [21] and visualized using UCSF Chimera [22].

### Purification of Maltose Binding Protein (MBP)-TcpF fusion protein and separation of TcpF from MBP

Since initial experiments to express TcpF in *E. coli* as a fusion protein with a poly-HIS tag at either the amino or carboxy terminus were not successful, we attempted purification of TcpF with a MBP tag, known to solubilize difficult proteins. Protein expression and purification utilizing an amylose-binding protocol was done as previously reported [23]. The *tcpF* gene from E99 was amplified and cloned into vector pMAL-c5E (New England Biolabs, Ipswich, MA) using the primers EF1959-F and EF1959-R, generating the plasmid pOU1811. For generation of the TcpF BB-loop mutant protein which does not contain the five amino acids DIFYS in the BB-loop region of Box 2, plasmid pOU1811 was used as template to conduct inverse-PCR amplification with the primers B2-F and B2-R. The PCR product was restricted with SalI, and self-ligated with Takara Solution I of the DNA Ligation Kit to generate plasmid pOU1812.

Purified pOU1811 and pOU1812 from transformed *E. coli* XL10-Gold cells were electroporated into expression host *E. coli* C41 (DE3) (Lucigen, Middleton, WI) to produce the wild type fusion protein MBP-TcpF and BB-loop mutant (MBP-TcpFm), respectively. Single colonies with plasmid containing pOU1811 or pOU1812 were inoculated into LB broth with glucose and ampicillin (100 µg/ml) and grown at 37°C with shaking. The culture was induced with isopropyl β-D-1-thiogalactopyranoside (IPTG) to a final concentration of 0.5 mM when the OD_600_ reached 0.6. The culture was then grown for 5 hours at 25°C. The purification of MBP-TcpF and MBP-TcpFm proteins was done according to the instructions outlined in the pMAL Protein Fusion & Purification System manual (New England Biolabs, Ipswich, MA). The MBP-TcpF fusion protein was digested by enterokinase (New England Biolabs, Ipswich, MA) using 1 ng enzyme per 25 µg protein, at room temperature for 16 h. To separate TcpF from MBP, the digested sample was diluted with binding buffer containing 25 mM MES, 25 mM NaCl (pH 5.75), and loaded onto a HiTrap Sepharose SP column. The column was then washed with 10 column volumes of binding buffer. Under these conditions, the MBP and any undigested fusion protein did not bind to the column and was collected in the pass-through and wash fractions. Bound TcpF was eluted using a 25 mM MES, 300 mM NaCl buffer and fractions checked on an SDS-PAGE gel followed by Coomassie blue R-250 staining. The MBP-TcpF fusion protein was used as an immunogen to raise polyclonal antibodies in rabbits (Proteintech Group Inc, Chicago, IL).

### Uptake of MBP-TcpF by RAW264.7 cells

RAW264.7 macrophages were seeded in 12-well plates, grown to confluence, and then inoculated with 10 µg/ml or 50 µg/ml purified MBP-TcpF or purified MBP control protein for 5 hours at 37°C. Cells were then washed five times with cold PBS and lysates prepared after treatment with trypsin at a final concentration of 0.25% for 5 minutes at 37°C. Proteins from cell lysate were resolved on a 10% SDS polyacrylamide gel and transferred to PVDF membrane. The membrane was blocked with 5% milk and probed first with anti-MBP monoclonal antibody (New England Biolabs, Ipswich, MA) overnight at 4°C followed by goat anti-mouse IgG conjugated to HRP. Bound antibody was detected using a chemiluminescent detection kit (Thermo Scientific, Rockford, IL).

### Analysis of PIP binding property of purified TcpF protein

Phosphatidylinositol phosphate (PIP) binding of MBP-TcpF fusion protein was evaluated as described earlier [19]. PIP strips (Echelon Biosciences, Salt Lake City, UT) were blocked with blocking buffer (10 mM Tris [pH 8.0], 150 mM NaCl, 0.1% Tween 20 and 0.1% ovalbumin) overnight at 4°C and then probed with purified MBP or MBP-TcpF fusion protein (0.5 µg/ml) at 4°C with gentle agitation. The membrane was sequentially incubated with anti-MBP monoclonal antibody and anti-mouse IgG-HRP. The bound protein was detected using SuperSignal West Pico Chemiluminescent Substrate (Pierce). To perform liposome pull-down assays, 200 µl of 1 mM PolyPIPosomes (Echelon Biosciences, Salt Lake City, UT) were mixed with 1 µg of TcpF or MBP protein in binding buffer (50 mM Tris [pH 8.0], 150 mM NaCl, and 0.05% Nonidet P-40) and rotated for 30 minutes at 25°C. The liposomes were washed three times with the binding buffer, boiled in sample buffer and analyzed by SDS-PAGE. The bound protein was detected by Western blotting.

### Immunoprecipitation

Recombinant MBP, MBP-TcpF or MBP-TcpF mutant (MBP-TcpFm) fusion protein purified from *E. coli*, was mixed with 1 mg RAW264.7 cell lysate overnight at 4°C under agitation. The mixture was precleared with protein A Sepharose beads (GE Healthcare, Piscataway, NJ) by rotation for 1 hour followed by incubation with anti-MBP antibody and protein A Sepharose beads for 4 hours. The immune complexes were collected by spinning at 500× g for 5 min. The beads were washed three times in lysate buffer before being subjected to polyacrylamide gel electrophoresis, followed by transfer to PVDF membrane for immunoblotting with MyD88 specific antibody (Cell Signaling Technology, Beverly, MA).

### Creation of TcpF mutant and complemented strains

The plasmid vector pLT06 was used to create an in frame markerless deletion of *tcpF* based on the protocol published by Thurlow *et al.* [24]. The regions 582 bp upstream and 814 bp downstream of *tcpF* were amplified by PCR using primer pairs EF1959-F/EF1961-R and EF1959-F1/EF1958-R, respectively, ligated to form a 1.4 Kb product and then inserted into pLT06. This construct was electroporated into *E. coli* strain EC1000 and grown on LB agar containing chloramphenicol and 5-bromo-4-chloro-indolyl-β-D-galactopyranoside (X-Gal). The colonies were then screened for the presence of the 1.4 Kb insert by PCR using primers Ori-F and KS05Seq-R which flank the MCS in pLT06. Positive colonies were selected and grown overnight in LB medium containing chloramphenicol (15 µg/ml) at 30°C. Purified plasmid (pSJP01) was verified by sequencing and then electroporated into *E. faecalis* strain E99. The selection of mutant clone was accomplished as described earlier [24], and labeled SPB03. The markerless gene deletion was complemented in *trans* by cloning *tcpF* along with its promoter region into plasmid pAT28. The tcpF gene along with its putative promoter region was amplified by PCR using primers EF1959-F and EF1958-R, and cloned as an EcoR1/BamH1 fragment into pAT28, generating pASB302. The plasmid pASB302 was electroporated into SPB03 and plated on BHI containing spectinomycin (500 µg/ml) to select the complemented strain labeled SPB04.

### Luciferase reporter assay

Mouse embryonic fibroblasts (MEF; 2×10^5^) were seeded on 24-well plates before transfection. NF-κB-luciferase reporter and Renilla luciferase reporter plasmids (Promega, Madison, WI) along with pCMV-TcpF (constructed by inserting the *tcpF* gene into the eukaryotic expression vector p3×FLAG-CMV-10), or the empty vector, were cotransfected into MEF cells using Lipofectamine 2000 (Invitrogen, Carlsbad, CA) according to the manufacturer's protocol. Cells were washed once with phosphate buffered saline (PBS) 24 hours later and then stimulated with LTA (5 µg/ml) for 8 h or TNF-α (20 ng/ml) for 5 hours. The samples were harvested in passive lysis buffer from the dual luciferase assay kit. After incubation at room temperature for 15 min, 25 µl of the lysate was assayed using the dual luciferase assay kit according to the suggested protocol. To monitor NF-κB activation during *E. faecalis* infection, MEF cells were transfected with NF-κB luciferase reporter and Renilla luciferase reporter plasmids using the above-mentioned method. After 24 hours of transfection, cells were incubated with strains E99, SPB03 and SPB04 at a multiplicity of infection (MOI) of 10 or LPS (0.5 µg/ml) for 5 hours, followed by harvesting in passive lysis buffer for assay according to the protocol outlined in the dual luciferase assay kit.

### Phagocytosis Assay

To determine the levels of phagocytosis of *E. faecalis* wild type strain E99, mutant strain SPB03 and complemented strain SPB04 by macrophages, 4×10^6^ CFU of bacteria were incubated with RAW264.7 macrophages in each well of a 24-well plate as previously described [25]. For conducting the phagocytosis assay, E99, SPB03 and SPB04 were transformed with plasmid pMV158GFP to constitutively express the green fluorescent protein. GFP-expressing strains were grown overnight at 37°C. The cells were centrifuged at 6000× g for 10 minutes and resuspended in Hank's balanced salt solution (HBSS). Subsequently, the macrophages were infected with the *E. faecalis* strains at an MOI of 10 and incubation carried out for 45 minutes at 37°C to allow bacterial uptake. Trypsin was then added at a final concentration of 0.25% for 10 minutes at 37°C to remove any residual bacteria at the macrophage cell surface. Cells were washed three times with PBS to remove remaining surface adherent bacteria and then subjected to flow cytometry using Accuri C6 flow cytometer (BD Accuri, Ann Arbor, MI).

### Bacterial survival within macrophages

Survival of *E. faecalis* within macrophages was assessed as described earlier [26], [27]. Triplicate wells of RAW264.7 macrophages were infected at an MOI of 100 for 1 hour at 37°C under 5% CO_2_. After infection, the cells were washed thrice with PBS and further incubated with DMEM supplemented with 10% FBS and containing vancomycin (16 µg/mL) and gentamicin (150 µg/mL) to kill all extracellular bacteria. At 2, 24, 48 and 72 hours, the macrophages were washed twice with PBS and harvested in 1 ml versene. The viability and cell count were assessed by Trypan blue staining using a TC10 Automated Cell Counter (Bio-Rad Laboratories, Inc., Hercules, CA). Macrophages were then lysed by adding one-tenth of the volume of a saponin cell lysis solution to release intracellular bacteria. Bacteria were quantified by serial dilution and plating. The number of viable bacteria at each time point was expressed as CFU per 10^5^ macrophages.

### Western blot

Following infection with *E. faecalis* wild type E99, TcpF mutant SPB03 or complemented strain SPB04 as described above, cells were washed with ice-cold phosphate-buffered saline (PBS) and placed on ice. The nuclear fraction was collected according to the protocol outlined in the Nuclear/Cytosolic Fractionation Kit (Cell Biolabs, San Diego, CA), and the protein concentration was determined using a Bio-Rad protein assay. The protein was subjected to polyacrylamide gel electrophoresis on 10% gels, and blotted onto a PVDF membrane which was then blocked with 5% skim milk. The PVDF membrane was incubated for 1 h with NF-κB-p65 or histone H4-specific antibodies in 3% skim milk. The blots were further incubated with appropriate secondary antibodies conjugated to horseradish peroxidase and developed using a chemiluminescent detection kit (Thermo Scientific, Rockford, IL).

The polyclonal antibodies (1∶1000) to TcpF, which were generated by immunizing rabbits with recombinant MBP-TcpF protein, was used to detect TcpF in cell lysates that had been resolved on 10% gels. Antibodies specific to rabbit IgG coupled with peroxidase (Cell Signaling Technology, Beverly, MA) served as secondary antibody (diluted 1∶3000). The membrane was developed using a chemiluminescent detection kit.

### Peritonitis model

All animal procedures were carried out in accordance with the guidelines set forth by the Public Health Service Policy on Humane Care and Use of Laboratory Animals. The University of Oklahoma Health Sciences Center Institutional Animal Care and Use Committee approved the protocol (10-173I) used in this work. Isoflurane was used for anesthesia/euthanasia and efforts were made to minimize suffering. *In vivo* studies were conducted as described earlier [28]. Bacteria were cultivated overnight at 37°C in THB containing 1% glucose supplemented with appropriate antibiotics. The cells were collected by centrifugation at 6,000× g for 10 min, washed twice in PBS (pH 7.4), and resuspended in 5% hog gastric mucin (pH 7.0; Pfaltz and Bauer, Waterbury, CT) at a concentration of 1.0×10^9^ CFU/ml. Six-eight week old female BALB/c mice (Harlan, Indianapolis, IN) were administered 200 µl of the suspension (2.0×10^8^ CFU) via intraperitoneal injection. Control mice were injected with 200 µl of 5% hog gastric mucin without bacteria to ensure both the sterility of the mucin and the absence of antibiotic-resistant bacteria in the tissues to be sampled. Control animals were treated in a manner identical to the experimental groups in all respects. At 24 h or 48 h postinfection bacteria in tissue homogenates were enumerated via serial dilution and plating as described earlier [27]. The histological examination and immunohistochemistry analysis of spleen and liver were performed as described earlier [29].

### Statistical analysis

Unless otherwise noted, the differences between groups were analyzed using Student's *t* test when only two groups were compared or using a one-way ANOVA when more than two groups were compared. The differences were considered statistically significant at *P*<0.05.

## Results

### Bioinformatics characterization, genomic location and protein structure of TcpF

**Figure 1:**
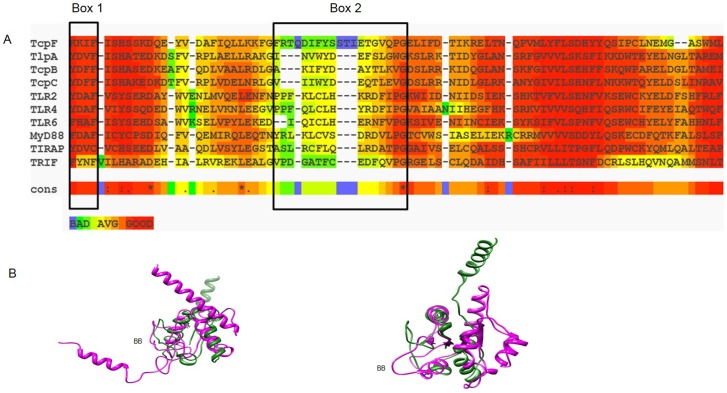
Comparison of TcpF to other known microbial TIR domain proteins and mammalian adaptor molecules. The top panel (A) shows alignment and domain organization of the predicted TIR domain of *E. faecalis* TcpF with representative TIR domains of bacteria (TlpA, TcpB, TcpC), human TLRs (TLR2, TLR4, TLR6) and adaptor molecules (MyD88, TIRAP, TRIF). Significant sequence similarity is observed in the Box 1 region with more sequence divergence evident in the Box 2 region, both of which are important signatures of all TIR domain proteins. The alignment was constructed with T-Coffee:: advanced server from EMBnet (http://www.ch.embnet.org/). The color scheme represented in the figure is indicative of the reliability of the alignment, with red corresponding to highest probability of correct alignment. Highly conserved identical residues including the glycine in the Box 2 region are denoted by an asterisk (*). (B) Superposition of the predicted TcpF-TIR (green) and *E. coli* TcpC-TIR (pink) domains (left) and TcpF-TIR (green) and human TLR2-TIR (pink) domains (right). The divergent BB-loop that defines specificity of TIR domain interactions is highlighted. Comparative structure modeling was done using 3D-JIGSAW for PDB generation and UCSF Chimera for visualization.

Employing a BLAST search approach we first analyzed the complete genome sequence of *E. faecalis* V583, the first vancomycin-resistant isolate in the US [30] for open reading frames (ORFs) that may encode homologs of human TIR domains. A single ORF, annotated as EF1959 and potentially encoding a 274 a.a. protein with a conserved TIR domain of unknown function, was identified. The EF1959 gene exhibits consensus upstream promoter elements and appeared to be expressed in isolation and not part of a polycistronic operon as verified by RT-PCR (Figure S1). A Position Specific Iterative BLAST (PSI-BLAST) analysis of EF1959 revealed a single domain at the N-terminus, conserved among members of the TIR superfamily in the Conserved Domain Database (CDD) [31]. The homology scores (E-value 3e-17) and identity length was in agreement with that observed for similar searches using peptide sequences corresponding to human adaptor proteins TIRAP, TRIF and TRAM. To further examine similarity, we aligned the TIR-domains from select TLRs, recently described bacterial Tcps and each of the known human adaptor proteins to that of the TIR-like domain of EF1959 (TcpF) using T-COFFEE [20]. As shown in Figure 1, there is significant overall homology between the TIR-like domain of TcpF and these other TIR domain-containing proteins. More importantly, the alignment also confirmed the presence of the Box 1 and Box 2 regions present in all bacterial Tcps and human adaptor proteins. While a high degree of conservation is evident for the Box 1 region, there is less overall sequence identity in the Box 2 region which includes the highly conserved glycine residue (Figure 1A). The Box 2 region is critical for TIR-TIR interactions [32] and it is to be expected that this region will exhibit variations in the amino acid residues comprising this region so as to maintain the specificity of interactions between interacting partners. Homology modeling and predicted 3-D structure comparisons further confirmed the similarities between TcpF and other TIR domain-containing proteins, all of which group to a single superfamily (3∶40∶50∶10140) in the CATH database of protein structures [33]. These analyses also revealed the functionally important BB loop extending as a distinct structure and positioned away from the core (Figure 1B). There was no evidence in TcpF of the less well conserved Box 3 region present in some TIR proteins [18]. Analysis of the carboxy terminus of TcpF extending from residues 240–274 revealed that it is enriched for polar and basic amino acids. Further, the isoelectric point for this region is high (9.61) compared to that for the rest of the protein (5.52). Interestingly this region also includes a basic amino acid motif (^240^ KVRFKLKKDK ^249^) similar to that identified in TcpB (^46^ KKRxxxxKK ^54^) which mimics TIRAP and shown to be important for binding to PIPs [19].

### Prevalence and genomic diversity

A search of the database comprising the high quality draft sequences and annotations of 16 *E. faecalis*, 8 *E. faecium*, 3 *E. casseliflavus* and 1 *E. gallinarum* strains by the Broad Institute [34], additional enterococcal genomes sequenced by Baylor College of Medicine and the Human Microbiome project, revealed that a single ORF with a 100% identity to EF1959 was also present in each of 22 unrelated *E. faecalis* strains, but not in any of the other enterococcal species. These results suggested that an EF1959-like protein was perhaps unique to *E. faecalis*, which is predominantly associated with enterococcal infections. In 3 other strains of *E. faecalis* (D6, OG1RF and T3) in the above databases, it appeared that genomic rearrangements in the region within/flanking *tcpF* (EF1959) might have occurred. We performed a PCR amplification using oligonucleotide primers that anneal to flanking ORFs EF1956 and EF1962 using genomic DNA as template. Agarose gel electrophoresis of PCR amplicons showed an expected product of ∼4.7 Kb in E99 (reference band), smaller amplicons of ∼4 Kb in D6 and T3 (suggesting deletion of at least a portion of EF1959) and a larger amplicon of ∼7 Kb in OG1RF (suggestive of DNA insertion at this locus) (Figure S2A). These PCR amplicons were sequenced, and the gene layout of region surrounding the open reading frame EF1959 showed that the strains D6, T3 and OG1RF have truncated EF1959 genes (Figure S2B). Transcriptional analysis by RT-PCR showed constitutive expression of EF1959 in *E. faecalis* V583 and E99 which were cultured in THB with 1% glucose, while a transcript was not detected in strain T3 (lacking the EF1959 gene) (data not shown). Analysis of ORFs annotated on the V583 genome both 5′ and 3′ to EF1959 showed multiple genes encoding proteins involved in carbohydrate and nucleotide metabolism, with some showing similarity to genes of phage origin, analogous to that observed for TlpA, TcpB and TcpC. Together, these data suggested that the genomic region encompassing *tcpF* might have been acquired by lateral transfer and be a hot spot for genomic recombination events.

### Purification and cell permeable property of TcpF

**Figure 2:**
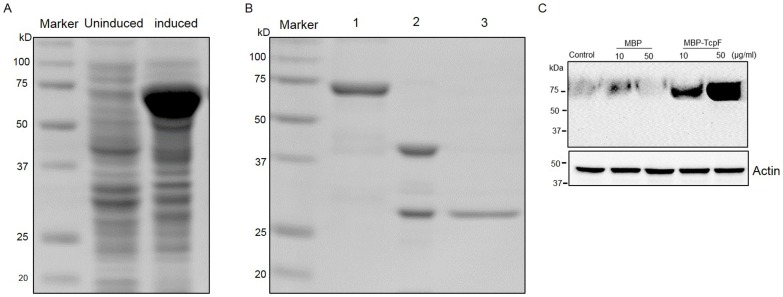
Expression, purification and characterization of MBP-TcpF. (A) Cell lysates from uninduced and induced cultures of C41 (DE3) *E. coli* harboring plasmid pOU1811 were separated on a 10% SDS-PAGE gel and stained with Coomassie blue R-250. (B) Purified MBP-TcpF (lane 1), MBP-TcpF digested with enterokinase (lane 2) and purified TcpF (lane 3) were run on a 10% SDS-PAGE gel and stained with Coomassie blue R-250. (C) RAW264.7 cells incubated with increasing concentrations of MBP-TcpF or MBP alone for 5 h. After washing and treatment with trypsin, the lysate was subjected to Western blot and probed with antibodies to TcpF.

Employing the pMAL-c5E vector, we were successful in purifying MBP-TcpF fusion protein that was expressed in the cytoplasm (Figure 2A). Efficient cleavage of the MBP tag was achieved by controlled digestion with recombinant enterokinase, and pure TcpF obtained by employing ion-exchange chromatography was checked on an SDS-PAGE gel following Coomassie Blue staining (Figure 2B). Identity was confirmed by limited N-terminal sequencing.

TIR domain-containing proteins in *Brucella* with a basic amino acid rich lipid-binding domain exhibited lipid-binding properties and cell permeability [19]. Since TcpF also harbors a similar region at the C-terminus, we wished to analyze the internalization of MBP-TcpF by eukaryotic cells. We incubated RAW264.7 macrophages with various concentrations of purified MBP-TcpF and analysis for translocated MBP-TcpF by Western blotting demonstrated that MBP-TcpF could be internalized by macrophages in a dose dependent manner. The internalization was not observed in cells incubated with MBP alone which indicated that the cell permeable property is attributable to TcpF (Figure 2C).

### TcpF can interact with phosphoinositides

**Figure 3:**
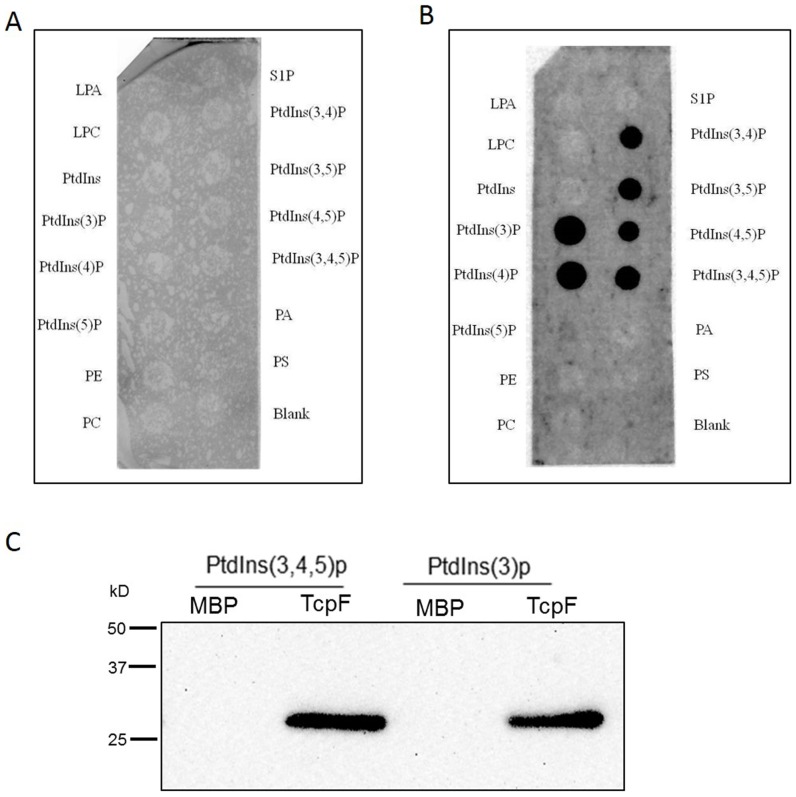
Analysis of the binding of TcpF to phospholipids. Western blot analysis of the interaction between various phosphorylated derivatives of phosphoinositol: (LPA, lysophosphatidic acid; LPC, lysophosphocholine; PtdIns, phosphoinositide phosphates; PE, phosphatidylethanolamine; PC, phosphatidylcholine, S1P, sphingosine-1-phosphate; PA, phosphatidic acid; PS, phosphatidyl serine) and MBP (A) or MBP-TcpF fusion protein (B) using PIP strip binding assay. (C) The binding of Ptdlns(3,4,5)p and Ptdlns(3)p to TcpF protein was confirmed by Poly PIPosome pull-down assay using anti-MBP-TcpF rabbit serum.

Phosphatidylinositol phosphate derivatives have been shown to be associated with TLR signaling and are predominant players in various other cellular signaling cascades [35]. A *Brucella* TIR domain-containing protein was found to interact with phosphatidylinositol derivatives and target TIR domain containing adaptor protein (TIRAP) mediated pathway to subvert TLR signaling [19]. An *in vitro* lipid-binding assay was therefore performed using a nitrocellulose membrane spotted with various phospholipids, to investigate the binding properties of MBP-TcpF with phosphoinositide derivatives. Protein overlay assays revealed no interaction between phosphoinositide derivatives and MBP (Figure 3A), whereas MBP-TcpF interacted with phosphatidylinositol 3-phosphate (PtdIns(3)P), phosphatidylinositol 4-phosphate (PtdIns(4)P), phosphatidylinositol 3,4-bisphosphate (PtdIns(3,4)P), phosphatidylinositol 3,5-bisphosphate (PtdIns(3,5)P), phosphatidylinositol 4,5-bisphosphate (PtdIns(4,5)P), and phosphatidylinositol 3,4,5-trisphosphate (PtdIns(3,4,5)P) (Figure 3B). Further confirmation of the specific interaction between TcpF and PIPs was evident from liposome pull-down assays (Figure 3C).

### Interaction of TcpF with MyD88 and inhibition of NF-κB activation induced by lipoteichoic acid

**Figure 4:**
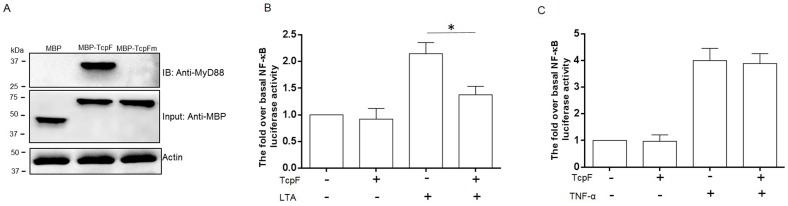
(A) Interaction between TcpF and MyD88. The immunoprecipitates obtained from incubation of MBP, MBP-TcpF or MBP-TcpFm (BB-loop mutant form) with lysates of RAW264.7 cells were analyzed by Western blot. (B & C) MEF cells were transiently transfected with NF-κB-luciferase and a plasmid expressing TcpF, and with pTK-Rel as an internal control. After 24 hours, transfected cells were stimulated with LTA (B) or TNF-α (C). Luciferase activity was measured and normalized to the activity of internal control, and fold activations relative to untreated samples were determined. Shown are averages and standard deviations of 3 independent experiments performed in triplicate. Significance * *P*<0.05.

To study the interaction between TcpF and the key adaptor protein MyD88, we expressed TcpF in *E. coli* as a fusion protein with an N-terminal MBP tag, and MBP-TcpF was used as bait protein with RAW264.7 macrophage cell lysate as prey. Immunoprecipitation analysis indicated a specific interaction between MBP-TcpF and MyD88 (Figure 4A). No interaction was detected between MBP and host cell MyD88. Because the BB-loop region from the TIR domain proteins are required for the two proteins to interact [36], we decided to investigate the effect of mutating the BB-loop region of TcpF, on this interaction. Accordingly, a MBP-TcpF mutant lacking the five amino acids DIFYS in the BB-loop region of Box 2 was generated and tested in the pull-down assays. As shown in Figure 4A, the BB-loop mutant of TcpF failed to interact with MyD88 thus confirming the importance of the BB-loop region in mediating the TcpF-MyD88 interaction.

To directly investigate the effect of *E. faecalis* TcpF on mammalian immune TLR signaling, the ORF encoding TcpF was amplified from *E. faecalis* E99 and cloned into the mammalian expression vector p3×FLAG-CMV-10. Using a NF-κB luciferase reporter based assay, the effect of TcpF on TLR signaling following exogenous stimulation was determined. While expression of TcpF had no significant effect on basal levels of reporter gene expression, in the presence of TcpF, LTA induced induction of NF-κB in MEF cells was decreased by 35% compared with the cells induced by LTA without TcpF (Figure 4B). In contrast, TcpF had no measurable effect on TIR-independent TNF-á-induced expression of NF-κB (Figure 4C). These results suggest that TcpF is acting specifically on TIR domain-dependent signaling.

### TcpF did not affect *in vitro* growth or phagocytosis by RAW264.7 macrophages

**Figure 5:**
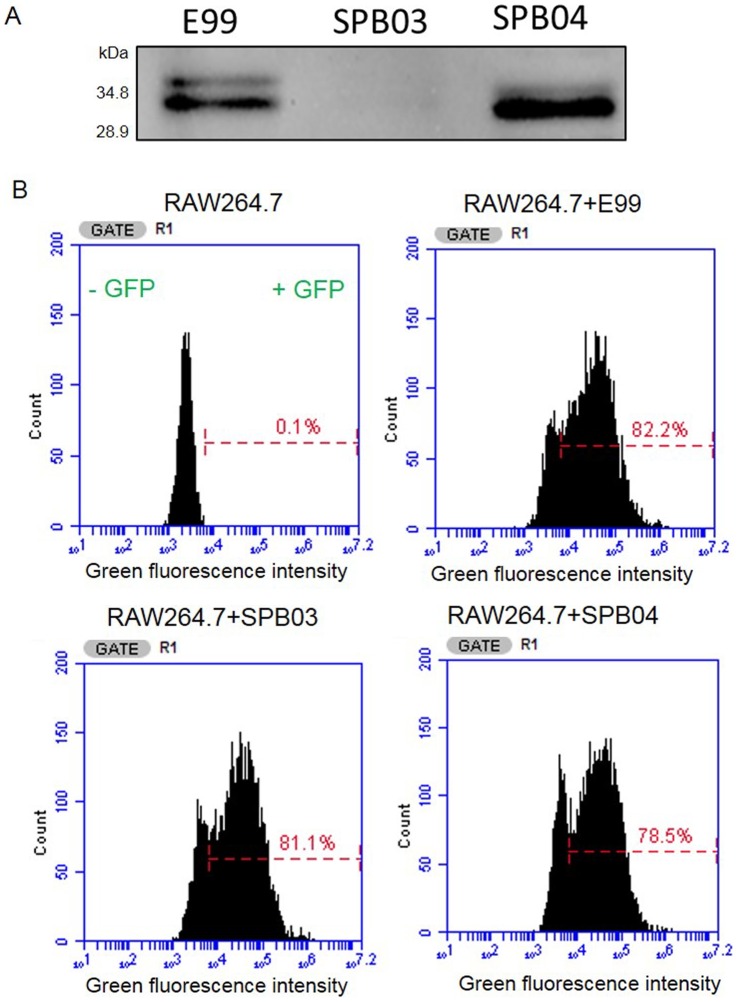
Characterization of TcpF wild type, deficient and complemented strains. (A) E99 was cultured in THB medium overnight and TcpF was detected in cell lysate by Western blot. The mutant and complemented strains were used as control. (B) Phagocytosis of *E. faecalis* by RAW264.7 cells. *E. faecalis* E99, SPB03 and SPB04 transformed with plasmid pMV158GFP were incubated with RAW264.7 cells for 45 minutes. Free bacteria were removed by washing and cells were analyzed by flow cytometry (BD Accuri, Ann Arbor, MI). These data are from a representative experiment that was repeated with similar results.

A mutant strain lacking the *tcpF* gene was created using homologous recombination in order to determine the role of TcpF during infection (Figure S3A & B). By generating a polyclonal antiserum to TcpF, we were able to detect TcpF in lysates of wild type E99 and complemented strain SPB04, but not in the lysates of TcpF mutant strain SPB03 (Figure 5A). Growth curves were obtained for the three strains of interest (E99, SPB03 and SPB04) to establish that the mutant strain was not compromised for growth under specified conditions in TSB supplemented with 0.75% glucose (Figure S3C) and THB supplemented with 1% glucose (data not shown) compared with the wild type and complemented strains. To measure phagocytosis by RAW264.7 cells, we employed GFP expressing E99, SPB03 and SPB04 and FACS analysis [25]. Our results showed that there was no significant difference in the number of macrophages infected with the three GFP^+^ strains (Figure 5B), thus confirming similar levels of phagocytosis.

### TcpF suppresses NF-κB activation during enterococcal infection

**Figure 6:**
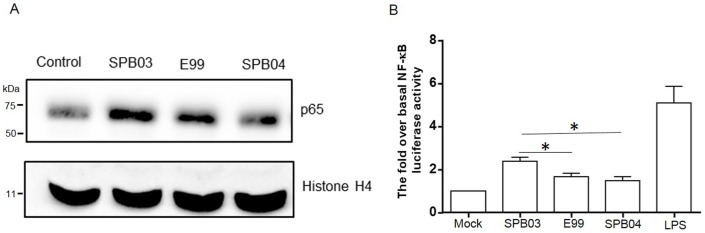
Role of TcpF in NF-κB activation. (A) The translocation of the p65 subunit of NF-κB to the nucleus of RAW264.7 macrophages infected (MOI = 10) with wild type, TcpF mutant (SPB03) and complemented strain (SPB04) for 1 hour or without infection (Control), was analyzed by subjecting the nuclear fraction to Western blot with p65 antibody. (B) The NF-κB transcriptional activity was assessed during infection of MEF cells by wild type, TcpF mutant and complemented strains. MEF cells were transfected with NF-κB-luciferase and Renilla-luciferase reporter constructs. After 24 hours, the medium was changed and cells were challenged with enterococci at a MOI of 10 or with LPS (0.5 µg/ml) as positive control. The data represent the mean values of three independent experiments and error bars indicate the standard deviations. Significance * *P*<0.05.

NF-κB is a transcription factor which is activated by Toll-like receptor signaling through TIR domain-containing adaptor molecules. To test the role of TcpF in NF-κB activation during enterococcal infection, we first compared the nuclear translocation of NF-κB-p65 (a subunit of the NF-κB transcription complex) during infection of mouse RAW264.7 macrophages with strains E99, SPB03 or SPB04, by Western blot. The results revealed that deletion of TcpF increases the abundance of p65 in the host nucleus during enterococcal infection compared with wild type and complemented strains (Figure 6A). To further confirm the role of TcpF in interfering with NF-κB activation, NF-κB reporter plasmid was used to transfect MEF cells to monitor the NF-κB transcriptional activation during enterococcal infection. The results from these experiments showed that the TcpF mutant SPB03 stimulated a much higher level of NF-κB activation compared to wild type and complemented strains (Figure 6B). Although the expression of TcpF did not totally abolish NF-κB activation, the diminished NF-κB activation during enterococcal infection may serve as one way to evade the host immune response.

### Effect of TcpF on the survival of *E. faecalis* in macrophages

**Figure 7:**
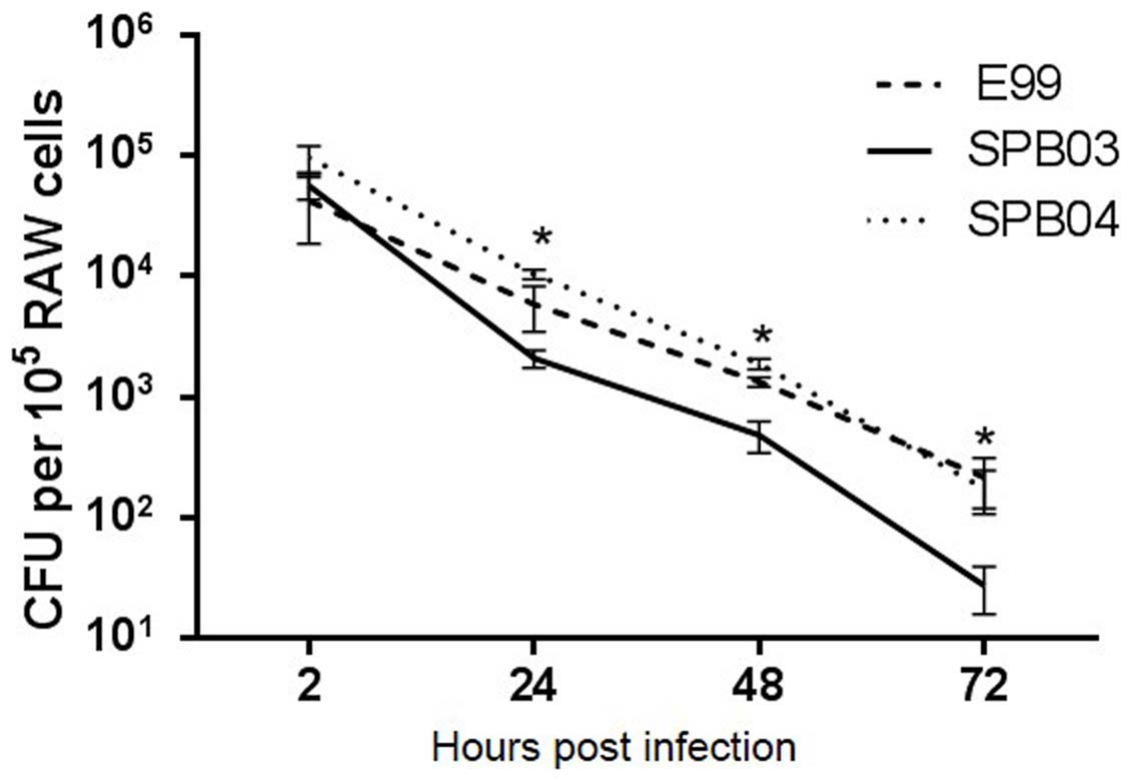
Characterization of intramacrophage survival of *E. faecalis* wild type (E99), TcpF mutant (SPB03) and complemented (SPB04) strains. Survival of E99, SPB03 and SPB04 based on colony-forming units per 10^5^ RAW264.7 macrophages, infected at a MOI of 100, after 2, 24, 48, and 72 hours post infection. Experiments were performed in triplicate. Significant differences (*P<0.05) were found at 24, 48, and 72 hours between wild-type and mutant.

The role of TcpF in host persistence was investigated using an *in vitro* macrophage survival assay [26]. Bacterial survival rates were compared for the internalized wild type, TcpF mutant and complemented strains in macrophages infected at a multiplicity of infection of 100. At 2 hours postinfection, all the strains reached a density of about 10^4^ CFU per 10^5^ macrophages. The number of viable bacteria for the wild-type and complemented strains is significantly higher than that of the TcpF mutant strain at 24, 48 and 72 hours postinfection (Figure 7). An approximately one log difference in bacterial numbers was observed between the wild-type and mutant at 72 hours postinfection.

### Role of TcpF in the virulence of *E. faecalis* in a murine peritonitis model

To examine the role of TcpF during *E. faecalis* infection, we evaluated the persistence of the bacteria and the degree of histopathology and neutrophil infiltration in organs, in a murine peritonitis model. No significant differences were observed between the persistence levels of wild type and the TcpF mutant strains at either of these time-points (Figure S4). There was also no significant difference in the severity of pathological changes in the liver and spleen between mice infected with the wild type or TcpF mutant strains (Figure S5). Although infiltration of neutrophils in spleens and livers was observed at 24 and 48 h post infection, no significant differences in the number of neutrophils in the liver and spleen between these two infected groups were observed (Figure S6). These observations suggested that TcpF is not a key element required for *E. faecalis* virulence in the mouse peritonitis model, under the conditions tested.

## Discussion

In our continuing efforts to identify novel virulence factors in the opportunistic pathogen *E. faecalis*, we have identified a protein with a TIR domain similar to that found in some other virulent bacteria, and in human adaptor proteins involved in the TLR signaling pathway. While initially identified in the genome of V583, the first infection-derived, vancomycin resistant *E. faecalis* isolate in the US, the analysis of other enterococcal genomes available in the various databases shows that the determinant encoding TcpF is not dispersed throughout the genus, but rather appears restricted to *E. faecalis* strains. With only a limited number of isolates screened so far, it is premature to draw definitive conclusions from these findings but nevertheless it is interesting that the determinant is absent in the *E. faecium* strains studied. In line with earlier reports which suggested that bacterial proteins encoding TIR domains are encoded in genomic regions of phage origins, we determined that *E. faecalis tcpF* is also located in a “hot spot” for recombination events [14]. Analysis of the regions surrounding the *tcpF* gene, revealed genes encoding some proteins of known function such as enolase and triosephosphate isomerase and some likely of phage origin. Further analysis of more number of diverse clinical and virulent strains will be necessary before definitive inferences can be made regarding the origin of *tcpF* in *E. faecalis*.

The deduced primary structure of TcpF indicates a hydrophobic protein, underscoring the challenges faced in overexpression and purification from *E. coli*. This is in agreement with observations for other bacterial TIR proteins such as TcpB and YpTdp which also required a solubilizing tag for efficient purification [19], [37]. The characteristics of TcpF precluded efficient expression of TcpF in *E. coli* by using a small tag such as a poly-HIS tag. The MBP tag is known to solubilize difficult proteins, and in this study was used to purify TcpF successfully. Although functional studies are best conducted with native protein devoid of extraneous tags, sometimes the study of native protein is limited by the lack of appropriate reagents such as antibodies. Epitope tags can often be used to label some proteins and to study the protein's biochemical properties. Some previous studies have shown that the MBP tag does not affect the structure and function of fused protein [38], [39]. Hence, in the studies described here we used TcpF fused with the MBP tag to investigate whether TcpF interacts with the host cell adaptor molecule MyD88. Consistent with the presence of the deduced BB-loop region, shown to be important for TIR-TIR interactions, we are able to show that MBP-TcpF can directly bind the adaptor molecule MyD88, while MBP alone or MBP-TcpF with a mutated BB-loop region of TcpF cannot do so. These results argue in favor of TcpF mediating the binding to MyD88.

It must be noted that for different adaptors to be recruited to the corresponding TLRs, there must be quite a degree of variation in the TIR domain of the TIR-containing protein which would satisfy the requirement of specificity in signal transduction. The conservation of TIR domain sequence has been demonstrated to be in the 20–30% range, with further variation in the size of the domains and associated conformational differences [40]. In accordance with this, in our study it is evident that the Box 2 region of TIR proteins show much less sequence conservation compared to the Box 1 region (Figure 1). The considerable variability in the Box 2 region is to be expected since it harbors the BB-loop region responsible for mediating the specific TIR-TIR interactions. It has also been reported that TIR domain sequence of some bacterial TIR proteins has a species-specific domain architecture [41].

As yet, the information regarding how bacterial TIR proteins localize to the host cell cytoplasm is quite limited. Studies to date indicate that different pathogens may use different mechanisms. It has been shown that the sequence of the region encoding TlpA in *S. enterica* is in proximity to determinants specifying proteins with pilin-related domains [14]. Since pilins play roles in cell adhesion as extracellular proteins, it was implied that proteins encoded within this genomic region including TlpA are secreted. However, no direct evidence for secretion of TlpA has been reported. Similarly, while it has been reported that TcpC is a secreted protein, this protein does not contain a recognizable signal sequence and the secretion mechanism is yet to be identified [15]. While we have no direct evidence to date to suggest that TcpF is secreted by *E faecalis*, it is important to consider this aspect in the context of how TcpF may gain access to the cytosol of host cells to mediate its interference with the TLR signaling pathway. As noted recently for the *Brucella* TcpB protein, *E. faecalis* TcpF also bound to PIPs immobilized on nitrocellulose or in pull-down assays. This lipid-binding property is presumably facilitated by the hydrophobic, cationic motif within the C-terminal domain of TcpF. Such endogenous cationic motifs have been shown to be important in proteins exhibiting cell permeable properties [17]. Indeed we have shown that tagged TcpF could be internalized by RAW264.7 cells (Figure 2). In this regard it is possible that the cationic C-terminal domain of TcpF may facilitate endocytosis by host cells.

Regardless of how TcpF gains access to the host cells, there remains the question of how TcpF is able to be recruited to the cell surface to interfere with TLR signaling. In mammalian cells, a defining feature of mammalian sorting adaptors such as MyD88 and TIRAP is their ability to localize to regions of the cell that contain TLRs even before signaling has been initiated [42]. This localization of sorting adaptor dMYD88 is facilitated by a ∼100 amino acid C-terminal extension consisting of polar residues and elevated isoelectric point in dMyD88, shown to be sufficient for membrane localization. In the case of TIRAP, a N-terminal domain consisting of residues 15–35 with a high isoelectric point (10.75) compared to the entire protein (7.55) that is enriched in basic and aromatic residues, was responsible for binding to PIPs [43]. In the present study, we found that TcpF could bind to phosphatidylinositol phosphates and interact with TIR adaptor protein MyD88. When we analyze TcpF for similar features to explain the binding to PIPs we find that the carboxy terminus extending from residues 240–274 is enriched for polar and basic amino acids, with a high isoelectric point (9.61) for this region compared to that for the rest of the protein (5.52), and includes the basic amino acid motif (^240^ KVRFKLKKDK ^249^) shown to be important for binding to PIPs [19]. Based on these observations we conclude that TcpF has a two-domain architecture and the PIP-binding domain at the C-terminus facilitates the N-terminal region harboring the TIR domain to interact with MyD88.

Another interesting consideration with respect to the PIP binding property of TcpF is the question of whether this may be playing a role in phagosomal maturation, fusion to lysosomes and killing of internalized enterococci in host cells. Previous studies have shown that phosphorylated derivatives of phosphatidylinositol are associated with endosome-to-lysosome trafficking, vesicle tethering, protein sorting, and modulation of various other cellular responses [35]. While enterococci are readily taken up by host phagocytes and the ability of enterococci to persist in infected host cells for extended periods is well documented [44], the mechanism for this has not been investigated in any detail. It is interesting to speculate that TcpF from internalized enterococci may serve to prevent proper targeting and display on the phagosomal membrane, of molecules such as PI(3)P, which are necessary for proper fusion to lysosomes and eventual elimination of phagocytized bacteria. While the validation of these hypotheses must await further studies, it nevertheless raises the possibilities of additional, hitherto unknown function(s) for TcpF and other bacterial TIR domain proteins.

Microbial infection often leads to activation of nuclear transcription factors such as NF-κB or AP1 resulting in a rapid and potent inflammatory response, as well as the regulation of antimicrobial effectors such as NADPH oxidase and inducible nitric oxide synthase which are important for elimination of intracellular bacteria [45]. However, a number of pathogenic bacteria have developed a variety of mechanisms to subvert host cell signaling to promote survival in these cells. An increasing number of studies have shown that bacterial TIR-domain proteins represent one such strategy to interfere with innate immune signaling affecting bacterial survival inside host cells. Studies with the TIR domain protein TcpC showed that it could directly bind to MyD88 and impede TLR signaling, thus facilitating intracellular survival of *E. coli* in RAW264.7 macrophages [15]. Consistent with this, we found in our study that *E. faecalis* TcpF could interference with the host cell TLR signaling pathway affecting activation of NF-κB and thereby promote survival in macrophages. The implication of enhanced survival in macrophages is important to consider in the context of *E. faecalis* which is a commensal organism of the GI tract. Translocation of *E. faecalis* across an intact intestinal epithelium has been demonstrated in the mouse and extraintestinal bacterial under these circumstances must be able to resist the microbicidal activity of tissue and peritoneal macrophages to disseminate and cause infection. By interfering with the TLR-signaling pathway through TcpF, *E. faecalis* may subvert the host immune response to enable its delivery from the intestinal lumen to draining mesenteric lymph nodes and thus be able to cause systemic infection.

The murine peritonitis infection model used to elucidate the immunomodulatory role of TcpF *in vivo* showed that there was no measurable effect of this protein on the virulence of this organism under the conditions tested, with respect to its ability to colonize and persist in host tissues to influence pathogenesis. It is quite possible that a higher or lower infectious dose, or assessment of bacterial burden and histopathology at other time points, may have revealed differences between the wild type and TcpF mutant in our peritonitis model. Interestingly, our observations are very similar to that noted for the TIR protein YpTdP which did not play a central role in the virulence of *Y. pestis* in a bubonic plague model, despite modulating TLR-dependent signaling *in vitro* [37]. This is not altogether surprising given the complex nature of pathogenesis and the limitations of *in vitro* assays in predicting the *in vivo* outcomes and relevant phenotypes associated with pathogenesis. In this regard, our results are also similar to those observed with other pathogens such as *Salmonella* spp., where a single assessment of virulence phenotype is not a good measure of the ability to cause disease or death in mice [46]. A recent study with the TirS protein from *S. aureus* has shown that the contribution of TIR proteins can vary significantly depending on the infection model used [18]. Enterococcal virulence may also be considered more subtle compared to that of overt pathogens and it is likely that TcpF must act in synergy with other virulence determinants to markedly influence enterococcal pathogenesis. Enterococcal genomes are extremely fluid with great diversity and it will take a careful examination of multiple virulent strains and various other infection models to recognize the precise roles and contributions of molecules such as TcpF to enterococcal pathogenesis.

In summary, our study has identified a TIR domain containing protein in *E. faecalis* that can effectively disrupt TLR-mediated signaling and interfere with NF-κB activation. The immunomodulatory effect of TcpF is dependent on its TIR domain. Further studies with TcpF, utilizing other models of infection are warranted to precisely define its role during infection.

## Supporting Information

Figure S1 Verification of transcripts for *EF1959*, *EF1958* and co-transcription of *EF1959-EF1958* in E99 and *tcpF*-deficient mutant, SPB03. Primer pairs EF1959-F2 and EF1959-R1 were used to amplify *EF1959*, EF1958-R1 and EF1958-F were used to amplify *EF1958*, and EF1959-F2 and EF1958-R1 were used to amplify *EF1959-EF1958*. Lanes 1–3 represent amplicons from *EF1959* (552 bp), *EF1958* (828 bp) and *EF1959-EF1958* (1759 bp) using E99 genomic DNA as template to validate the primers. Lanes 4-15 represent reaction products from E99 and SPB03 templates. (+) denotes cDNA template and (−) denotes mRNA control (minus reverse transcriptase) to show samples were free of contaminating genomic DNA.
(TIF)

Figure S2 Diversity in the chromosomal location of the EF1959 locus encoding TcpF. (A) Genome diversity in the region of EF1959 (ORFs EF1956 to EF1962) is indicated by varying size of the PCR amplicons obtained from DNA of *E. faecalis* E99 (reference band) and three other unrelated strains D6, OG1RF, T3. M: 1 Kb size marker. (B) Comparison of the chromosomal organization of the region flanking EF1959 in strains V583, E99, D6, T3 and OG1RF. Annotations based on V583 genome: EF1956- hypothetical protein; EF1958- deoxyguanosinetriphosphate triphosphohydrolase-like protein; EF1959- hypothetical protein; EF1961- Enolase; EF1962- triosephosphate isomerase.
(TIF)

Figure S3 Generation of TcpF-deficient *E. faecalis*. (A) Schematic of the creation of the TcpF mutant strain by homologous recombination. (B) Relative size of amplicons from wild type (WT), single cross over (SCO) and double cross over (DCO) mutants. (C) *In vitro* growth curves for E99, SPB03, and SPB04. Growth curve was generated from bacteria grown at 37°C in TSB supplemented with 0.75% glucose. Experiments were performed in triplicate and no significant differences among the three strains were apparent.
(TIF)

Figure S4 Bacterial burden in mice infected with *E. faecalis* wild type (E99) and TcpF mutant strain (SPB03). Mice (n = 9) were challenged by the peritoneal route with 2×10^8^ CFU of either wild type or TcpF-mutant strain. At 24 (A) and 48 (B) hours following infection, blood, spleen and liver were collected and bacterial numbers were enumerated by serial dilution and plating. A non-parametric Mann Whitney test was utilized to determine significance (*P*<0.05) levels.
(TIF)

Figure S5 Histopathology of mouse tissues following infection with *E. faecalis* E99 wild type (WT) and TcpF mutant strain (SPB03). Mice were challenged by the peritoneal route with 2×10^8^ CFU of either wild type or TcpF mutant. At 24 and 48 hours after infection a portion of the spleen (A) and liver (B) was stained with hematoxylin and eosin (H&E) and evaluated. Organs from 3 animals were examined and representative data are shown here. Cellular degeneration and necrosis in the liver and a significant depletion of the white pulp along with the number of lymphocytes decreasing in the spleen are evident.
(TIF)

Figure S6 Neutrophil infiltration in mice infected with *E. faecalis* wild type or TcpF mutant strains. The spleen (A) and liver (B) from mice either uninfected (Control), infected with *E. faecalis* wild type (WT) or infected with TcpF mutant (SPB03) strains were harvested at 24 hours or 48 hours post infection and stained with rabbit anti-neutrophil elastase.
(TIF)
