# Supplementary figures and images for: A TIR Domain Protein from E. faecalis Attenuates MyD88-Mediated Signaling and NF-κB Activation
Source: PLoS One. 2014 Nov 4;9(11):e112010. doi: 10.1371/journal.pone.0112010 (PMC4219826; doi:10.1371/journal.pone.0112010)

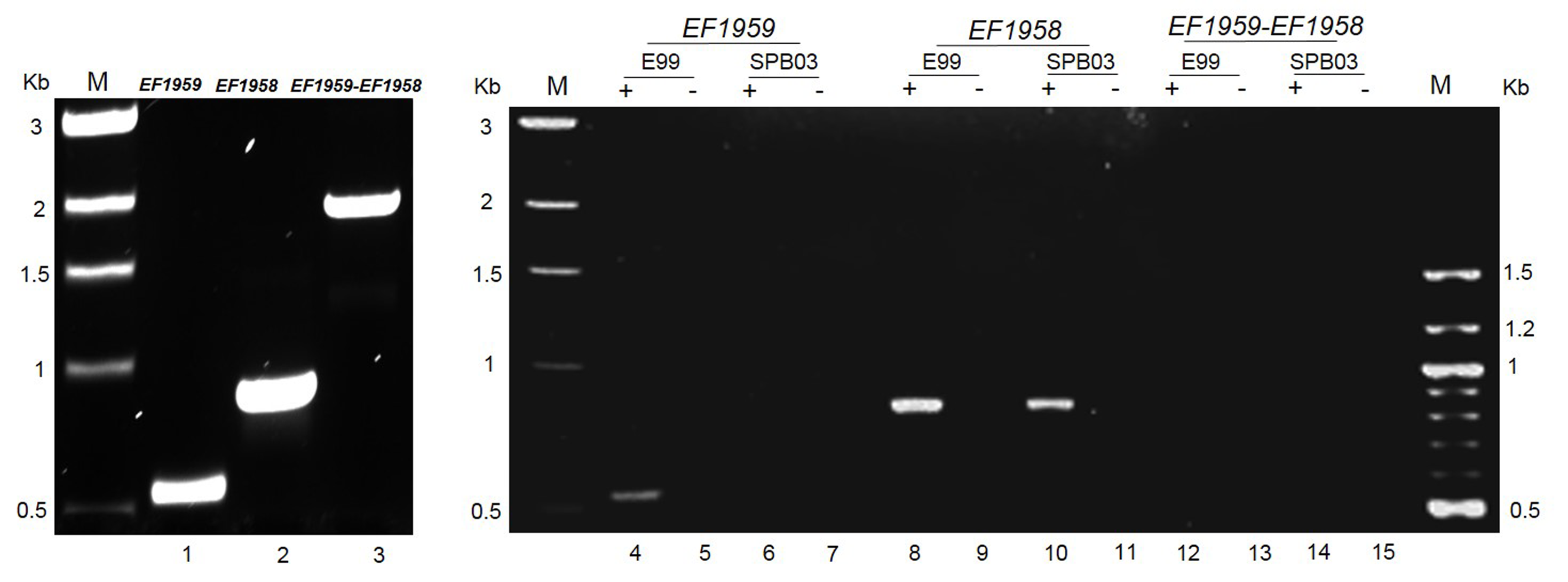

Supplement: Figure S1 — Verification of transcripts for EF1959, EF1958 and co-transcription of EF1959-EF1958 in E99 and tcpF-deficient mutant, SPB03. Primer pairs EF1959-F2 and EF1959-R1 were used to amplify EF1959, EF1958-R1 and EF1958-F were used to amplify EF1958, and EF1959-F2 and EF1958-R1 were used to amplify EF1959-EF1958. Lanes 1–3 represent amplicons from EF1959 (552 bp), EF1958 (828 bp) and EF1959-EF1958 (1759 bp) using E99 genomic DNA as template to validate the primers. Lanes 4-15 represent reaction products from E99 and SPB03 templates. (+) denotes cDNA template and (−) denotes mRNA control (minus reverse transcriptase) to show samples were free of contaminating genomic DNA. (TIF) [file pone.0112010.s001.tif]

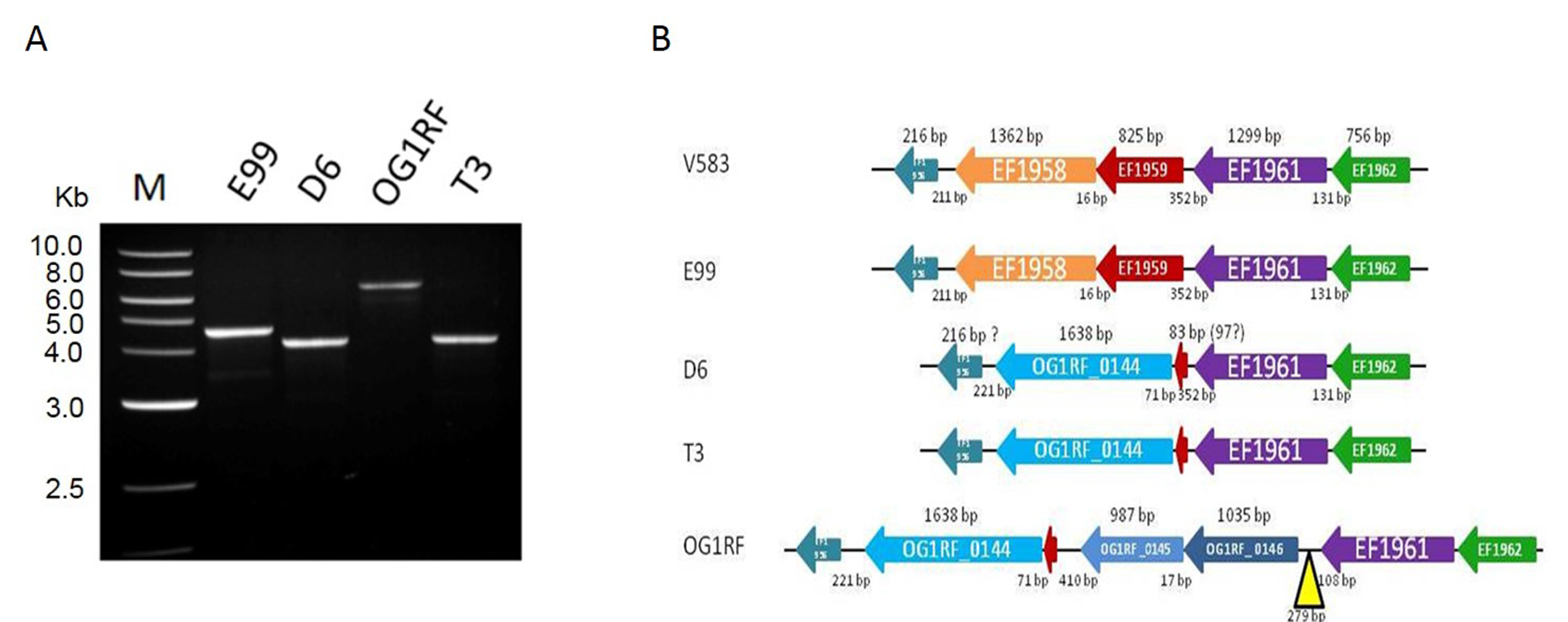

Supplement: Figure S2 — Diversity in the chromosomal location of the EF1959 locus encoding TcpF. (A) Genome diversity in the region of EF1959 (ORFs EF1956 to EF1962) is indicated by varying size of the PCR amplicons obtained from DNA of E. faecalis E99 (reference band) and three other unrelated strains D6, OG1RF, T3. M: 1 Kb size marker. (B) Comparison of the chromosomal organization of the region flanking EF1959 in strains V583, E99, D6, T3 and OG1RF. Annotations based on V583 genome: EF1956- hypothetical protein; EF1958- deoxyguanosinetriphosphate triphosphohydrolase-like protein; EF1959- hypothetical protein; EF1961- Enolase; EF1962- triosephosphate isomerase. (TIF) [file pone.0112010.s002.tif]

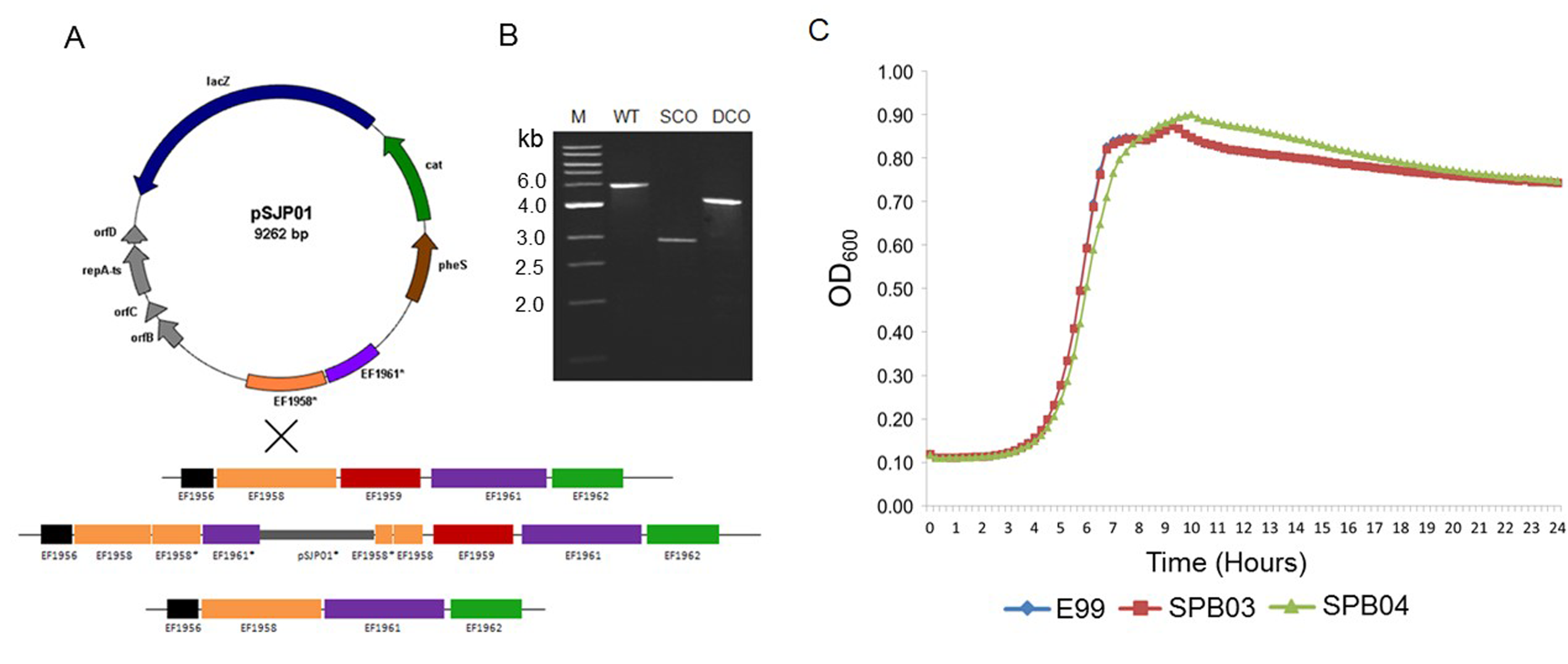

Supplement: Figure S3 — Generation of TcpF-deficient E. faecalis. (A) Schematic of the creation of the TcpF mutant strain by homologous recombination. (B) Relative size of amplicons from wild type (WT), single cross over (SCO) and double cross over (DCO) mutants. (C) In vitro growth curves for E99, SPB03, and SPB04. Growth curve was generated from bacteria grown at 37°C in TSB supplemented with 0.75% glucose. Experiments were performed in triplicate and no significant differences among the three strains were apparent. (TIF) [file pone.0112010.s003.tif]

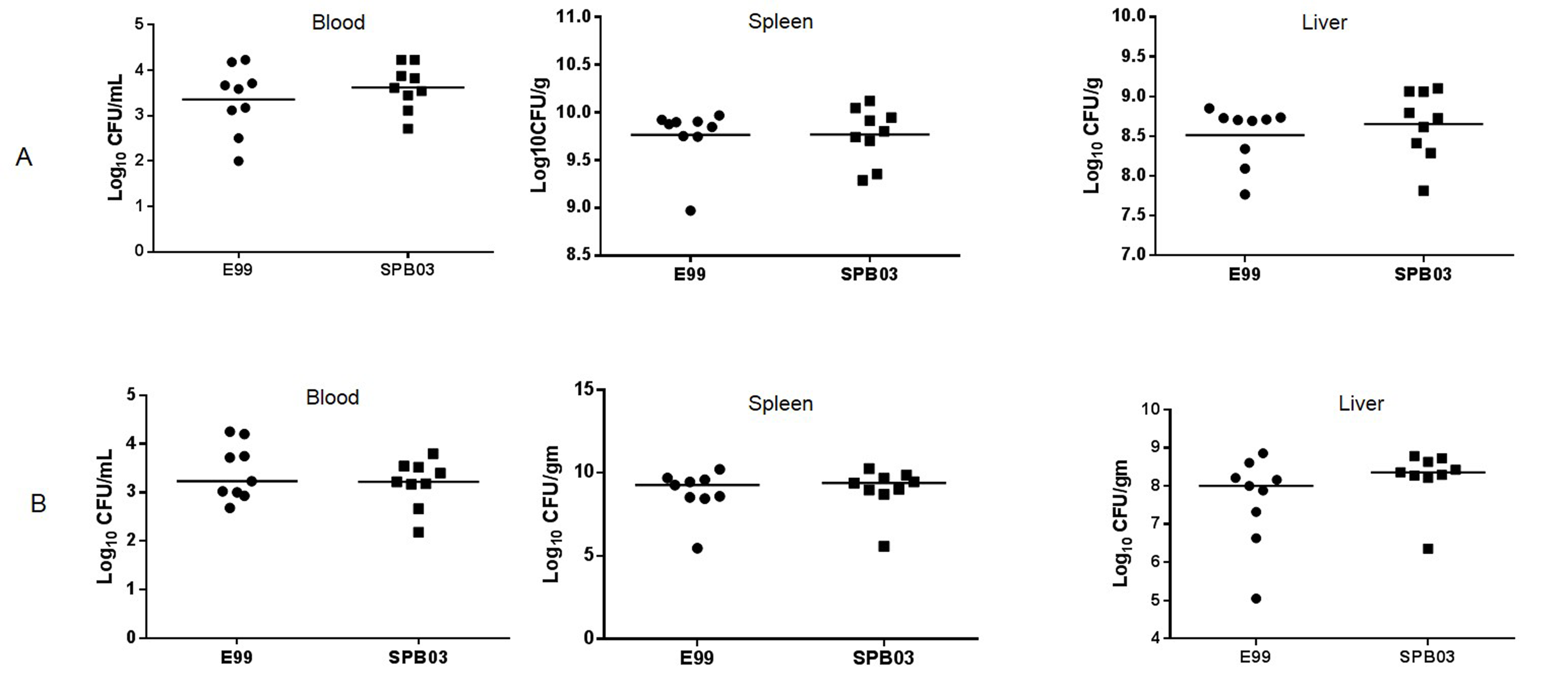

Supplement: Figure S4 — Bacterial burden in mice infected with E. faecalis wild type (E99) and TcpF mutant strain (SPB03). Mice (n = 9) were challenged by the peritoneal route with 2×10^8^ CFU of either wild type or TcpF-mutant strain. At 24 (A) and 48 (B) hours following infection, blood, spleen and liver were collected and bacterial numbers were enumerated by serial dilution and plating. A non-parametric Mann Whitney test was utilized to determine significance (P<0.05) levels. (TIF) [file pone.0112010.s004.tif]

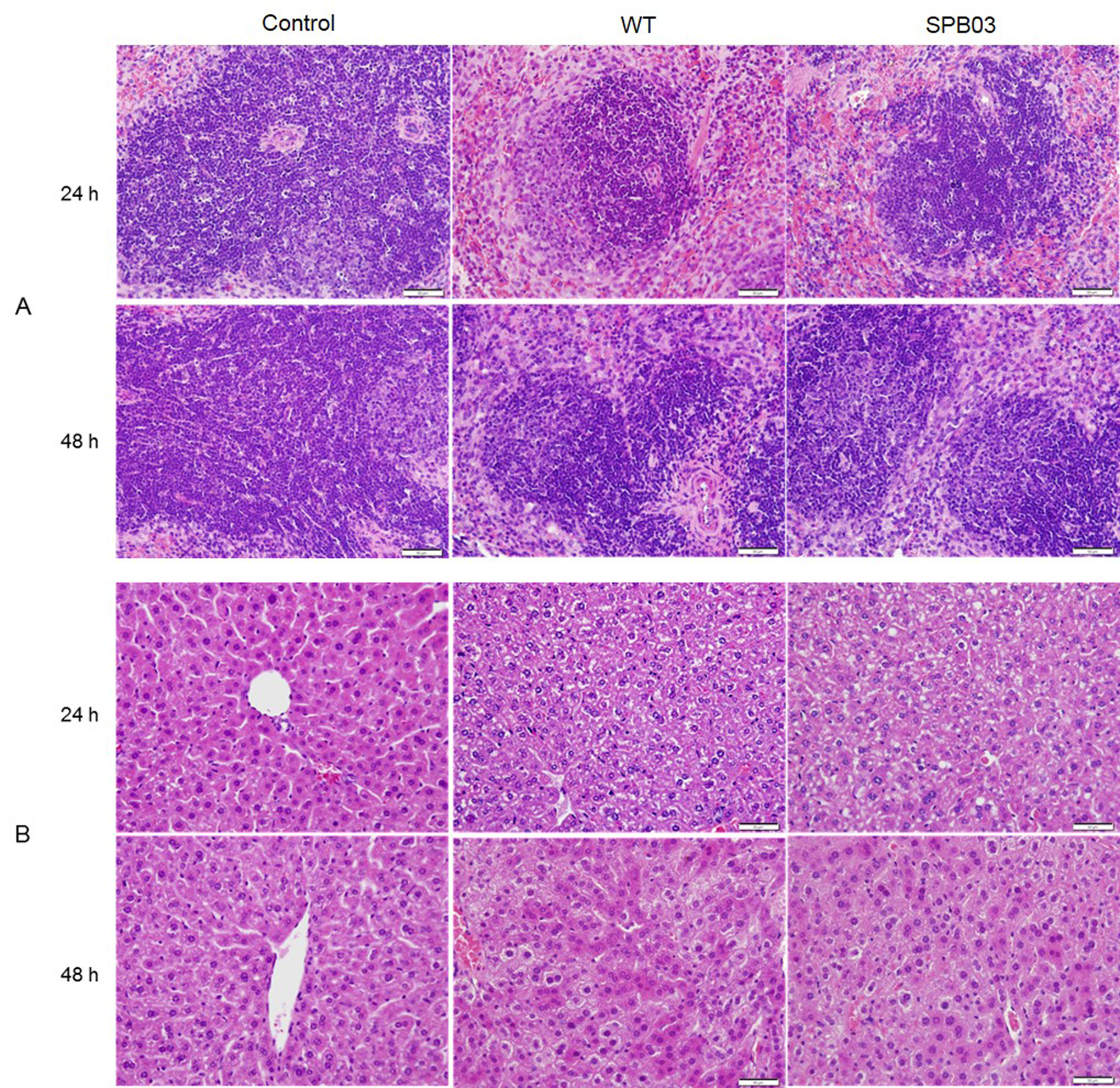

Supplement: Figure S5 — Histopathology of mouse tissues following infection with E. faecalis E99 wild type (WT) and TcpF mutant strain (SPB03). Mice were challenged by the peritoneal route with 2×10^8^ CFU of either wild type or TcpF mutant. At 24 and 48 hours after infection a portion of the spleen (A) and liver (B) was stained with hematoxylin and eosin (H&E) and evaluated. Organs from 3 animals were examined and representative data are shown here. Cellular degeneration and necrosis in the liver and a significant depletion of the white pulp along with the number of lymphocytes decreasing in the spleen are evident. (TIF) [file pone.0112010.s005.tif]

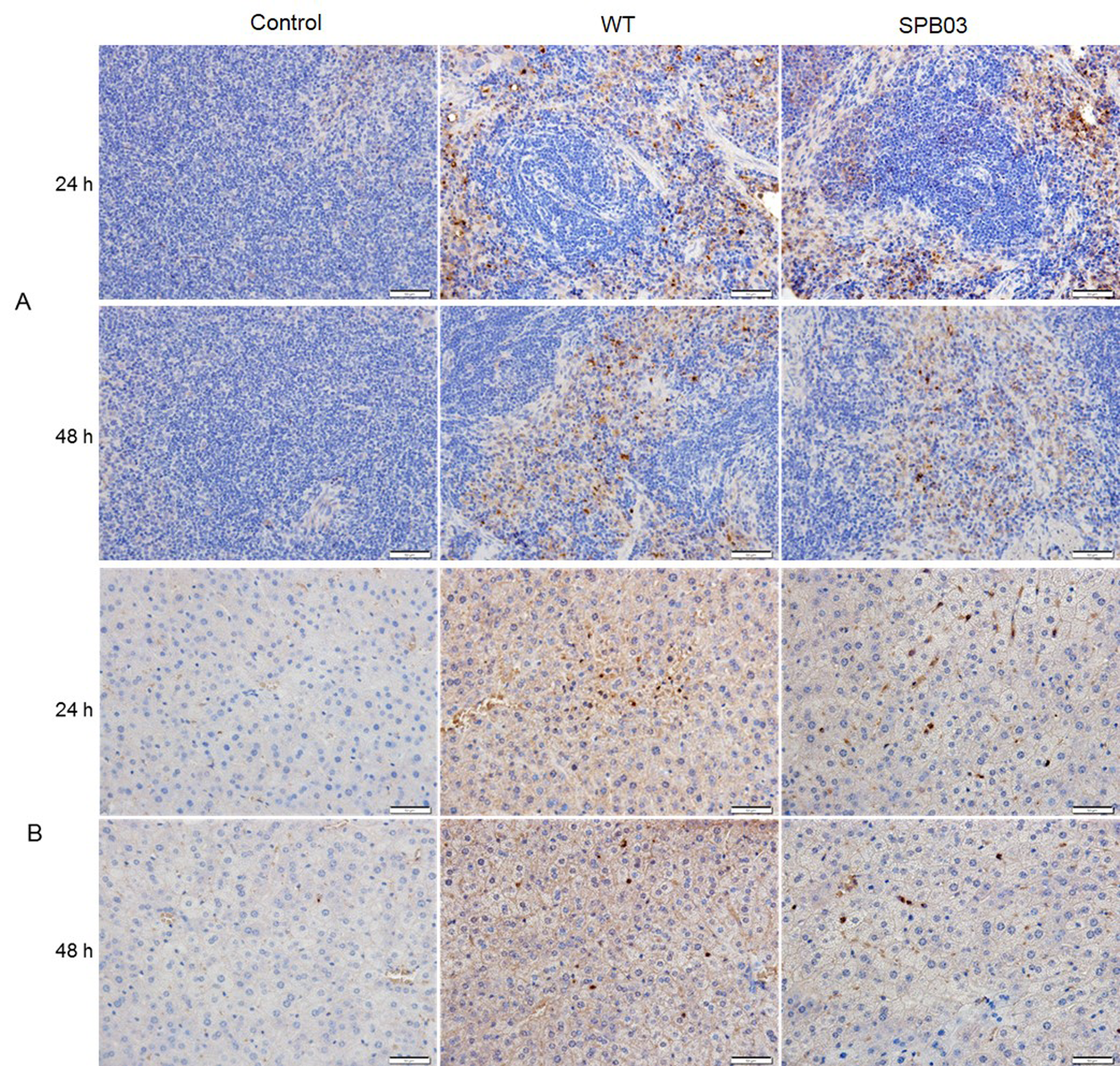

Supplement: Figure S6 — Neutrophil infiltration in mice infected with E. faecalis wild type or TcpF mutant strains. The spleen (A) and liver (B) from mice either uninfected (Control), infected with E. faecalis wild type (WT) or infected with TcpF mutant (SPB03) strains were harvested at 24 hours or 48 hours post infection and stained with rabbit anti-neutrophil elastase. (TIF) [file pone.0112010.s006.tif]
